# 

**Published:** 1902-04

**Authors:** 


### PERSONALS.

Prof. Leonard Pearson, Dean of the Veterinary Department, University of Pennsylvania, entertained Captain Kendall, of the Veterinary Department of the English army, located at Melbourne, Australia, at dinner at the University Club of Philadelphia and afterward at a theatre party, in February.

Dr. F. A. Wall, M.R.C.V.S., formerly of Pittsburg, Pa., is in the artillery service of the British army in South Africa. He has been confined to the hospital of the army nursing a serious injury received from the kick of a horse.

Dr. E. D. Barnsdale, of Jackson, Mich., is fond of speedy and well-bred horses. Besides being the owner of the well-bred stallion "Young Wayne," he is also the possessor of a fast pacer.

Dr. T. E. Gore, formerly of Buffalo, N. Y., and a former assistant of Dr. John Wende, is now a resident of Clarksburg, W. Va., where he conducts a livery stable in connection with his practice.

Dr. Leon H. Reefer, of Wheeling, W. Va., was the victim of a rabid dog bite in December, and spent some weeks in New York undergoing the Pasteur treatment, from all of which we are glad to report his complete recovery.

